# The effect of natural adjuvants (G2, G2F) on lung inflammation of sensitized guinea pigs

**Published:** 2013

**Authors:** Ali Neamati, Mohammad Hossein Boskabady, Saleh Mohaghegh Hazrati, Mohammad Reza Khakzad, Shakeeb Hassan Moosavi

**Affiliations:** 1 *Department of Biology, Faculty of Science, Islamic Azad University, Mashhad Branch, I. R. Iran*; 2 *Applied Physiology Research Centre andDepartment of Physiology, School of Medicine, Mashhad University of Medical Sciences, Mashhad, I. R. Iran*; 3 *School of Public Health, Tehran University of Medical Sciences and Researches on Industrial Biotechnology, Tehran, I. R. Iran*; 4 *Islamic Azad University, Mashhad Branch, and Zakaria Reserch Center, Mashhad, I. R. Iran*; 5 *Faculty of Medicine, NHLI, Imperial College, London, UK*

**Keywords:** Asthma, Natural adjuvant, Sensitized animals, WBC

## Abstract

**Objective:** The effects of natural adjuvants were examined on total and differential WBC counts in lung lavage of sensitized guinea pigs.

**Materials and Methods:** In three sensitized groups of guinea pigs including: untreated sensitized animals (S), sensitized animals treated with adjuvant G2 (S+G2) and G2F (S+G2F) as well as non-sensitized group (C) (n=6 for each group), total and differential WBC counts of lung lavage were examined. Sensitization of animals was achieved by injection and inhalation of ovalbumin (OA).

**Results:** The results showed increased total WBC, eosinophil, neutrophil, and basophil counts, and decreased lymphocytes in lung lavage of sensitized animals compared with the control group (p<0.01 for all cases). However, neutrophil, lymphocyte, eosinophil, and basophil counts in lung lavage were decreased in treated groups with either G2 or G2F but total WBC was decreased in lung lavage of treated group with only G2.

**Conclusion:** These results indicate important preventive effects of two natural adjuvants, especially G2, on lung inflammation of sensitized guinea pigs.

## Introduction

Asthma is an inflammatory disorder of the airways (Busse et al., 1995 ▶) and its main characteristic is airway hyperresponsiveness (AHR) (Padrid et al., 1995 ▶). G2 is the buffalo spleen extract. It was shown that consumption of spleen extract regulate the immune system (Greer, 1932 ▶; Gray, 1933 ▶) by inhibiting lymphocyte proliferation in tissue-specific (Heidemann et al., 1979) and stimulating polymorphonuclear leukocyte activities (Krasowski et al., 1992).

G2F adjuvant is the concentrated G2 adjuvant in sesame seed oil. It was reported that this oil increases vaccine potency (Fukumi, 1976), cellular immunity as an adjuvant (Kimura et al., 1978b), and natural killer cell activity (Pope et al., 1992), but not affect antibody production (Kimura et al., 1978a). Moreover, the antioxidant activity and lipid peroxidation inhibitory effect of sesame seed were also documented (Niwa et al., 1988; Fazel et al., 2009).

The effect of G2 adjuvant on breast cancer (Mohaghegh et al, 1999 ▶) and its stimulatory effect on Th-1 cells have also been observed (Mohaghegh et al, 2006 ▶) and its therapeutic effects on different illnesses such as allergy and asthma are also suggested (Mohaghegh et al, 2004 ▶). The preventive effects of G2 and PC (another adjuvant of bacterial origin) on sensitized guinea pigs have been also been demonstrated previously (Neamati et al, 2009 ▶). Therefore, in the present study, we examined the effects of the G2 and G2F adjuvants on total and differential WBC counts in bronchoalveolar lavage (BAL) of sensitized guinea pigs.

## Materials and Methods

**Preparation of the adjuvants**

G2 adjuvant was prepared from buffalo spleen lipid and registered as a patent in the Iranian Patent Office as: Immune System Activator Vaccine (Innovation Register No: 36679, 28th of October 2006). Briefly, buffalo spleen was crushed in small pieces and diluted in alcohol for a few days, before being centrifuged at 800× g for 30 min. The supernatant was dried to gain a concentration of 20 µg/ml. G2 adjuvant components include different types of lipids, glucose, cholesterol, and triglyceride.

For G2F preparation, crude sesame seed oil from Esfahan province (located in the middle of Iran) was filtered with 0.5 µm filter paper to remove particles. The oil was then centrifuged at 10000 rpm at room temperature. The supernatant was filtered with 0.22 µm pure size filters under vacuum filtration. Three hundred µg/ml of G2 was added to the resulting preparation called G2F and divided into 5 ml aliquots in glass vials under sterile conditions. 

**Animal sensitization and different groups**

Four groups of Dunkin-Hartley guinea pigs (550 to 700 g, purchased from Razi Animal Institute, Mashhad, Iran, n=6 for each group) were studied. The four groups were: 1) non-sensitized, control group (C), 2) animals sensitized to ovalbumin (an animal model of asthma), which received normal saline, 0.5 ml (i.p.) twice a week for 4 weeks (S), 3) sensitized animals treated with adjuvant G2, 0.4 ml (i.p.) twice a week for 4 weeks (S+G2), and 4) sensitized animals treated with adjuvant G2F, 0.1 ml (i.p.) twice a week for 4 weeks (S+G2F), (Mohaghegh et al, 1999; Neamati et al, 2009). 

The animals were group-housed in individual cages in climate-controlled animal quarters and given water and food ad libitum, while a 12-h on/12-h off light cycle was maintained. Guinea pigs were sensitized to OA (Sigma Chemical Ltd., UK) by injecting 100 mg (i.p.) and 100 mg (s.c.) on day one and a further 10 mg (i.p.) on day 8. From day 14, sensitized animals were exposed to an aerosol of 4% OA for 18±1 days, 4 min daily (McCaig and Jonckheere, 1993; Boskabady et al., 2006). Control animals were treated similarly but saline was used instead of OA solution. The aerosol was administered in a closed chamber (dimensions: 30×20×20 cm^3^). The study was approved by the ethical committee of the Mashhad University of Medical Sciences.

**Lung lavage and its white blood cells count**

After sacrificing guinea pigs, their lungs were lavaged with 20 ml of DulBecco’s phosphate-buffered saline which was aspirated after gentle lung massage. Bronchoalveolar lavage (BAL) fluid was collected and centrifuged at 2500 rpm for 10 min (at room temperature) and the supernatant was collected. Precipitated cells were pulled on slides, fixed, and stained with Wright-Giemsa. Differential cell counts were done using standard morphologic criteria and eosinophil, neutrophil, lymphocyte, and basophil were identified under a light microscope and their numbers were expressed as the percentage of 400 cells counted. Total white blood cell (WBC) count was performed by Neubauer lam using torch stain using non-centrifuged BAL.

**Statistical analysis**

The data were quoted as mean±SEM. According to the Kolmogorov Smirnov test, these data had normal distribution. The data of four groups of animals were compared using ANOVA test. Significance was accepted at p<0.05.

## Results

**White blood cell count**

**Figure 1:**
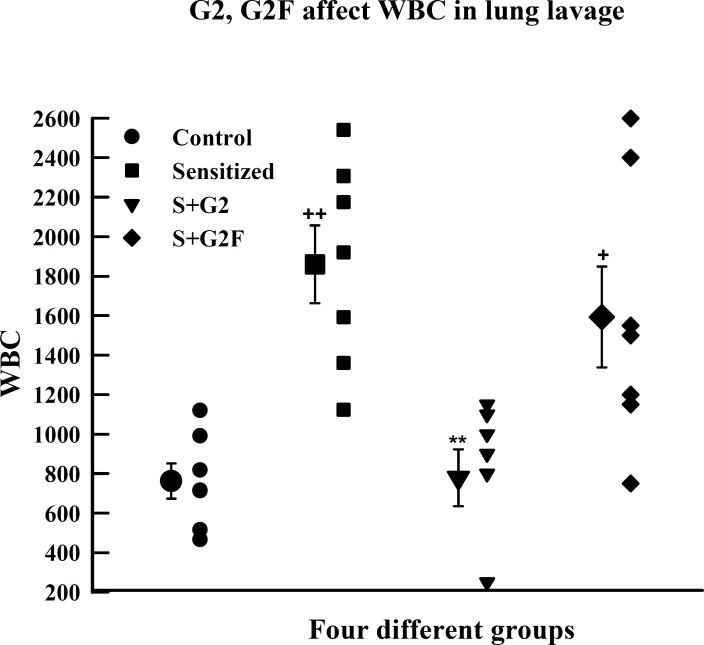
Individual values and mean±SEM (big symbols with bars) of total white blood cell count in four groups of guinea pigs (n=6): control (C), sensitized (S), S treated with adjuvant G2 (S+G2), and S treated with adjuvant G2F (S+G2F). Statistical differences between control and other groups: +; p<0.05, ++; p<0.01. Statistical differences between G2 and G2F *vs.* S group: **; p<0.01

The mean value of total white blood cell (WBC) in lung lavage of S animals (1860±197) was significantly higher than that of control group (762±90, p<0.01). However, treatment of S animal with S+G2 caused a significant reduction in total WBC (779±143, p<0.01) (Figure 1). 

**Differential count of WBC in lung lavage fluid**

There was a significant increase in the percentage of eosinophils, neutrophils, and basophils and a decrease in percentage of lymphocytes (p<0.001 for all cases) in lung lavage fluid of S animals compared with those of controls. All changes in eosinophil, neutrophil, basophil, and lymphocyte seen in S group were reversed in both treatment groups which were statistically significant for all changes except for basophil in S+G2F group (p<0.001 for all cases, Figure 1). However, the percentage of eosinophils in S+G2 and basophils in S+G2F groups were still significantly different from those of control group (p<0.05 and p<0.001, respectively) (Figure 1). 

**Differences in total and differential WBC counts between two** **groups of animals treated with the adjuvant**

The values of total WBC count and percentage of basophils in lung lavage in S+G2F group were significantly higher than those of S+G2 group (p<0.05 to p<0.001, Table 1). 

**Table 1:** Values of total and differential count of WBC in lung lavage of control, sensitized (S), S treated with adjuvant G2 (S+G2), and S treated with adjuvant G2F (S+G2F) guinea pigs (n=6 for each group) and statistical differences between S+G2 and S+G2F groups

**Cell Type**	**Control **	**S**	**S + G2**	**S + G2F**
**Total WBC** **Eosinophil** **Neutrophil** **Lymphocyte** **Basophil** **Monocyte**	762.14±89.59 8.71±1.85 16.29±1.50 46.00±2.96 4.43±0.92 11.86±1.32	1859.64±197.06 28.57±0.92 34.43±1.50 23.29±1.86 13.64±0.79 15.21±9.96	778.57±143.45 14.43±1.70 15.43±1.88 41.85±2.52 6.14±1.18 12.00±1.63	1592.86±255.28 ¶ 15.14±0.83 ¶¶ 14.00±1.68 49.43±1.13 9.57±0.72 13.57±0.65

Values are quoted as mean±SEM. Units of total WBC is the number of cells in 1 ml of lung lavage and units of each cell type is its percentage of all cell types. Statistical *significance* for the *difference* between the data of S+G2 vs. S+G2F:

¶ ; p<0.5,

¶¶ ; p<0.01.

**Figure 2:**
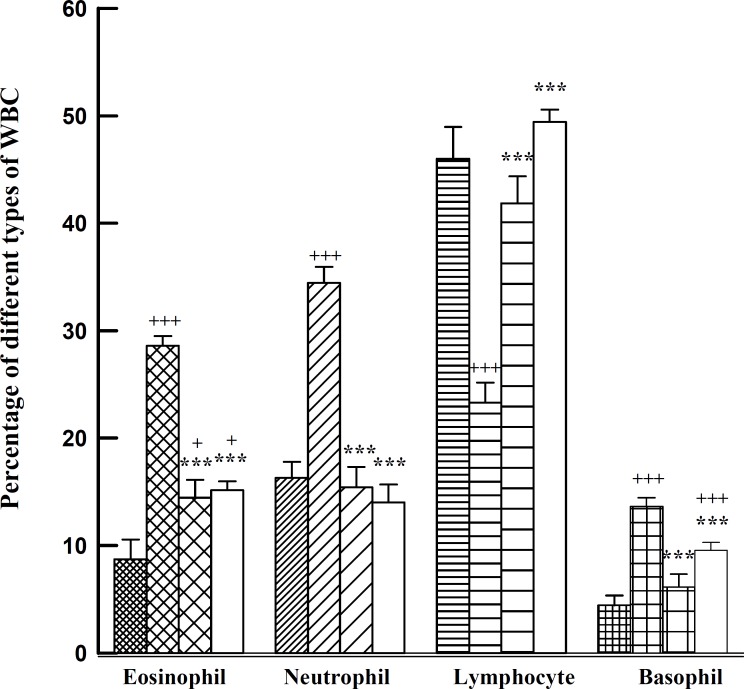
The percentages of eosinophil, neutrophil, lymphocyte, and basophil of lung lavage in control (fine filled bars, C), sensitized (medium filled bars, S), S treated with adjuvant G2 (coarse filled bars, S+G2), and S treated with adjuvant G2F (open bars, S+G2F), (n=6 for each group). Statistical differences between control and other groups: +; p<0.05, +++; p<0.001. Statistical differences between S+G2 and S+G2F *vs.* S group: ***; p<0.001

## Discussion

In the current study, increased total WBC number in sensitized guinea pigs compared with control animals was shown. The percentages of eosinophil, neutrophil, and basophil were also increased but lymphocyte decreased in S animals compared with those of control group. Pretreatment of S animals with adjuvants G2 or G2F inhibited or diminished the changes in eosinophil, neutrophil, basophil, and lymphocyte. The G2 adjuvant also inhibited the changes in total WBC and the effect of the G2 adjuvant on total WBC and eosinophil counts in sensitized animals was greater than the effect of G2F adjuvant except for the change in lymphocytes.

Inhibitory effect of G2 or G2F on increased leukocytosis and eosinophilia in sensitized guinea pigs indicates that the studied adjuvants have anti-inflammatory properties. The possible mechanism for the effects of the adjuvants relates to their antioxidant properties. Both enzymatic and nonenzymatic antioxidant defenses were reduced in asthma (Sackesen et al., 2008). Oxidative stress plays an important role in asthmatic airway inflammation and may be a useful therapy for bronchial asthma (Cho YS et al., 2004 ▶). 

While therapeutic effects of dietary antioxidants in asthma is contraversial (Romieu et al., 2002 ▶; Trenga et al., 2001 ▶), a relationship between dietary interventions, oxidative stress, bronchial inflammation, asthmatic symptoms severity, and lowered cellular reducing capacity was reported (Trenga, 2001 ▶). Supplementation with an antioxidant inducer (Ayurvedic health food supplement =Amrita Bindu) to asthmatic patients stopped asthma attacks and the need to use anti-asthmatic medications (Kumar and Shanmugasundaram, 2004). A-Lipioc acid was also suggested as a useful adjuvant therapy for bronchial asthma (Cho YS et al., 2004 ▶). Antioxidant supplementation also led to a reduction in the dose of corticosteroids required by asthmatic patients (Gvozdjáková et al., 2005). Therefore, the mechanism of action of studied adjuvants on sensitized animals could be due to their antioxidant effect. In our previous studies, we have shown that vitamin C, an antioxidant agent, has a relatively powerful preventive effect on increased tracheal responsiveness of sensitized guinea pigs using a similar method of sensitization (Boskabady and Ziaei, 2003 ▶). We have also previously shown that an extract of Nigella sativa, which also has antioxidant properties, has a preventive effect on asthma (Boskabady et al., 2007 ▶). 

The extract of sesame seed has been shown to increase natural killer cell activity (Kimura et al., 1978b) and cellular immunity (Fukumi, 1976) and has antioxidant activity (Niwa et al., 1988; Fazel et al., 2009). Therefore, the protective effect of the extract of sesame seed could be due to its effect on immunity mechanisms or its antioxidant property. Since the natural adjuvants used in the current study affected total WBC, especially eosinophils, and because eosinophils produce several oxidant agents, it may indicate that these adjuvants have antioxidant effect. 

The results of the present study showed that the preventive effect of treatment with G2 adjuvant was higher than that of the G2F adjuvant on total WBC and eosinophil counts. In addition, the administered dose of the G2F form of the adjuvant was higher than the G2 adjuvant (30 µg *vs.* 8 µg) and the extract of sesame seed existing in G2F adjuvant itself showed cellular immunity and antioxidant properties. However, the preventive effect of G2 on increased eosinophil and neutrophil was non-significantly greater than G2F, but this effect on total WBC and basophil was statistically significant. The mechanism of these findings is unclear and should be examined in further studies.

Regarding the safety of these adjuvants, the effects of more than 10000 injections in rabbits, guinea pigs, and BALB/c mice with different doses, different intervals, and combinations of adjuvants (unpublished data) were examined and no side effect was observed. In addition, the dose and duration of adjuvant administered in this study were much lower than those mentioned above. The results of this study suggest a preventive effect of G2 and G2F adjuvants on asthma disease by reducing total and differential WBC of sensitized animals. However, their effect and optimum dose of administration in asthmatic patients and their tolerance should be examined in further studies.

In conclusion, the results of the present study indicated preventive effects of G2 and G2F natural adjuvants on changes of total and differential WBC of lung lavage. The preventive effect of G2 on lung inflammation was greater than G2F adjuvant. 

## References

[B1] Boskabady MH, Javan H, Sajadi M, Rakhshandah H (2007). The possible prophylactic effect of Nigella sativa seed extract in asthmatic patients. Fundament Clin Pharmacol.

[B2] Boskabady MH, Kiani S (2007). The effect of expouser of guinea pigs to cigarette smoke and their sensitization with ovalbumin in tracheal responsiveness to histamine and hisatamine (H1) receptor blockade by chlorpheniramine. Pathophysiology.

[B3] Boskabady MH, Ramazani M (2001). Relaxant effect of Pimpinella anisum on isolated guinea pig tracheal chains and its possible mechanism(s). J Ethnopharmacol.

[B4] Boskabady MH, Ziaei T (2003). Effect of ascorbic acid on airway responsiveness in ovalbumin sensitized guinea pigs. Respirology.

[B5] Boskabady MH, Kiani S, Aslani MR (2006). Tracheal responsiveness to both isoprenaline and beta2- adrenoreceptor blockade by propranolol in cigarette smoke exposed and sensitized guinea pigs. Respirology.

[B6] Busse W, Banks-Schlegel SP, Larson GL (1995). Childhood versus adult-onset asthma. Am J Respir Crit Care Med.

[B7] Cho YS, Lee J, Lee TH (2004). Alph-Lipoic acid inhibits airway inflammation and hyperresponsiveness in a mouse model of asthma. J Allergy Clin Immunol.

[B8] Fazel M, Sahari MA, Barzegar M ( 2009). Comparison of tea and sesame seed oils as two natural antioxidants in a fish oil model system by radical scavenging activity. Int J Food Sci Nutr.

[B9] Fukumi H ( 1976). Experience of nasal application of inactivated influenza vaccine. Dev Biol Stand.

[B10] Gray GA (1933). The treatment of agranulocytic angina with fetal calf spleen. Texas State J Med.

[B11] Greer AE (1932). Use of fetal spleen in agranulocytosis: preliminary report. Texas State J Med.

[B12] Gvozdjáková A, Kucharská J, Bartkovjaková M (2005). Coenzyme Q10 supplementation reduces corticosteroids dosage in patients with bronchial asthma. Biofactors.

[B13] Heidemann E, Podgornik N, Wilms K (1979). Tissue-specific inhibitor of lymphocyte proliferation extracted and purified from calf spleen. Biol Chem Blut.

[B14] Kimura J, Nariuchi H, Watanabe T (1978a). Studies on the adjuvant effect of water-in-oil-in-water (w/o/w) emulsion of sesame oil Enhanced and persistent antibody formation by antigen incorporated into the water-in-oil-in-water emulsion. Jpn J Exp Med.

[B15] Kimura J, Nariuchi H, Watanabe T (1978b). Studies on the adjuvant effect of water-in-oil-in-water (w/o/w) emulsion of sesame oil. 2. Mode of action of the w/o/w emulsion. Jpn J Exp Med.

[B16] Krasowski H, Stehle P, Fürst P (1992). The effect of the calf spleen and calf thymus extracts, thymopentin and tuftsin, on the phagocytosis activity of neutrophilic granulocytes. Arzneimittelforschung.

[B17] Kumar SS, Shanmugasundaram KR (2004). Amrita Bindu-an antioxidant inducer therapy in asthma children. J Ethnopharmacol.

[B18] McCaig D, De Jonckheere S (1993). Effect of two Ca2+ modulator in normal and albumin sensitized guinea-pig trachea. Eur J Pharmacol.

[B19] Mohaghegh Hazrati S, Aghazadeh J, Mohtarami F (2006). Immunotherapy of Prolactinoma with a T Helper 1 Activator Adjuvant and Autoantigens: A Case Report. Neuroimmunomodulat.

[B20] Mohaghegh Hazrati S, Khanbaba CR, Mazaheri B (1999). A novel an invasive metastatic Adenocarcinoma of breast cancer (HAZ-1) in small with laboratory mice. 14th Iran Con Physiol Pharmacol.

[B21] Mohaghegh HS, Nasiri Khalaji S, Mohtarami F (2004). G2, PC and G2F in allergic asthma. Iran J Ped.

[B22] Neamati A, Boskabady MH, Tavakol Afshari J (2009). The effect of the natural adjuvants on tracheal responsiveness, and cell count of lung lavage in sensitized guinea-pig. Respirology.

[B23] Niwa Y, Kanoh T, Kasama T (1988). Activation of antioxidant activity in natural medicinal products by heating, brewing and lipophilization A new drug delivery system.. Drugs Exp Clin Res.

[B24] Padrid P, Snook S, Finucane T (1995). Persistent airway hyperresponsiveness and histologic alteration after chronic antigen challenge in cats. Am J Respir Crit Care Med.

[B25] Pope BL, Chourmouzis E, Sigindere J (1992). In vivo enhancement of murine natural killer cell activity by 7-allyl-8-oxoguanosine loxoribine. Int J Immunopharmacol.

[B26] Romieu I, Sienra-Monge JJ, Ramirez-Aguilar M (2002). Antioxidants supplementation and lung function among asthmatic children exposed to high levels of air pollutants. Am J Respir Crit Care Med.

[B27] Sackesen C, Ercan H, Dizdar E (2008). A comprehensive evaluation of the enzymatic and nonenzymatic antioxidant systems in childhood asthma. J Allergy Clin Immunol.

[B28] Trenga CA, Koenig JQ, Williams PV (2001). Dietary antioxidants and ozone-induced bronchial hyperresponsiveness in adults with asthma. Arch Environ Health.

